# Transcriptome using Illumina sequencing reveals the traits of spermatogenesis and developing testes in *Eriocheir sinensis*

**DOI:** 10.1371/journal.pone.0172478

**Published:** 2017-02-17

**Authors:** Gen-Liang Li, Hui Qian

**Affiliations:** Youjiang Medical University for Nationalities, Baise, Guangxi, China; Zhejiang University College of Life Sciences, CHINA

## Abstract

Chinese mitten crab (*Eriocheir sinensis*) has the spermatozoa with typical aflagellate, decondensed chromatin, cup-shaped nuclei, and radial arms. However, the mechanism of spermatogenesis during which the specific spermatozoa are generated in this species is yet unclear. Here, the transcriptome of developing testis in *E*. *sinensis* was analyzed using the ways of RNA-seq and bioinformatics analysis to identify candidate genes potentially involved in development of testis and spermatogenesis. The Illumina HiSeq2500 sequencing of three replicons of samples produced a total of 145.19 M clean reads representing with a total of 21.34 Gb bases and 45.48% GC content. 56.30% clean reads were mapped to the draft genome of *E*. *sinensis*. The assembly of the transcriptome yielded contigs of 5691802 sequences and unigenes of 406527 sequences. Total 24246 and 40793 transcripts were annotated using Swissprot and Nr database, respectively. There were 48213 (70.31%) and 7858 (46.25%) transcripts with identity of more than 99 matching to mature testis unigenes in the databases of Nr and EST, respectively. The analytic results of KOG, GO and KEGG showed wide potential molecular functions of transcripts in the developing testes. KEGG analysis of unigenes yielded total 9422 predicted genes. Those predicted genes were involved in total 216 KEGG pathways related to the physiological activities of developing testis. 1975 predicted genes were involved in cellular and subcellular structural alteration of male germ cells. There were important roles of some pathways in the processes of morphological and structural biogenesis pertaining to testis development and spermatogenesis. Other 583 unigenes encoding the genetic and epigenetic factors also be found, which might contribute to the decondensation and stability of decondensed nuclei in the spermatozoa. These predicted events provide a view of the potential molecular mechanisms of development of testis and spermatogenesis in *E*. *sinensis*.

## Introduction

Chinese mitten crab (*Eriocheir sinensis*), an economically important aquaculture animal in China, has the spermatozoa with typical aflagellate. More interestingly, the genetic material in spermatozoal nuclei is decondensed, the spermatozoal nuclei are cup-shaped, and there are about 20 radial arms derived from the nuclear membrane on the surface of each spermatozoon [1]. Therefore, there would be a unique mechanism participated in the regulation of spermatogenesis in this type of species. So the physiological characteristics of testis during the spermatogenesis of *E*. *sinensis* is an active research area.

To date, we have clearly known the structure and the spatiotemporal changes of the male reproductive system and the various germ cells in the male reproductive system during the spermatogenesis [1–3]. In recent 2 decades, researchers have been exploring the possible mechanisms of spermatogenesis at the molecular level [4–10]. In recent years, with the rapid development of high-throughput sequencing technologies, we have been able to explore the gene expressing patterns and the possible physiological or pathological function in the specific tissues and organs in a particular developmental stage or under the condition of influence of specific environmental factors at the fully transcriptomic level. Recently, the transcriptomic traits and possible gene expression information of mature testis in *E*. *sinensis* have been studied using the techniques of RNA-seq and de novo assembly [11–13]. In those studies, a number of potentially functional genes and pathways related to spermatogenesis and testis development were found, such as ERK1/ERK2 MAPKs, Rho, Rac, Cdc42, cadherin and PAR3, DDX51, DDX55, Dbp73, ubiquitin mediated proteolysis pathway, and so on. The probably roles of those predicted genes and pathways in reproduction of *E*. *sinensis* were also discussed, such as the functions of cell cycle proteins, actin, DEAD box family genes involved in spermatogenesis, ubiquitin-dependent proteolytic system, anti-hyperthermia stress, and anti-oxidative stress genes in the testis. However, the developing testis and the types of reproductive cells in the developing testis of immature crab were different from ones of mature crab. Consequently, the transcriptome of mature crab testis mainly reflects the possible gene expressing patterns of mature testis, especially predominant spermatozoa which are deposited in the testis. Therefore, we should study the traits of transcriptome of developing testis in *E*. *sinensis* to explore its possible patterns of gene expression for drawing the intrinsic genetic programing involving the process of testis development and the spermatogenesis in this species.

In this study, the transcriptome of developing testis in *E*. *sinensis* was sequenced using RNA-seq technology, and mapped to the draft genome of *E*. *sinensis* recently assembled [14]. To date, there is no fully sequenced genome of this species and its current draft genome can only provide us with about 60% of the genomic sequences of the species. Therefore, we also assembled the sequencing reads using de novo to obtain unigenes and functionally annotated the unigenes using the Nr, KOG, Swissprot, KEGG, GO database. It can let us get as comprehensive information as possible at present for understanding the traits of the transcriptome of developing testis at the developmental stage of testis in *E*. *sinensis*. The results could help us to better the understanding of the gene expression patterns and the possible mechanisms of spermatogenesis in the developing testis of *E*. *sinensis* at the transcriptome level.

## Materials and methods

### Animals

The developing males of *E*. *sinensis* at the stage of spermatid, wet weight of each body is 6.0–8.0g, were collected from a commercial aquafarm in Ganyu County, Jiangsu Province, China in March 2014. The individuals were placed in an ice bath for about 2 min until they were lightly anesthetized. Testes of sixty male were removed surgically and washed with PBS, and then immediately immerged in RNA guarder (Waryong, Beijing, China) and stored at -20℃ until required. The male germ cells in those testes consist of a large number of spermatids and some other cells such as spermatogonia, primary spermatocytes, secondary spermatocytes, and spermatozoa. Sixty Testes were divided into 3 replicons (XC-1, XC-2, and XC-3). Each twenty random testis tissue were pooled as one sample for RNA extraction. As for use of *E*. *sinensis*, no approval is needed due to it is a common, economical, and edible animal in China.

### RNA extraction and cDNA library preparation

Each sample was used for total RNA extraction with TRIzol reagent (Invitrogen, Shanghai, China) according to the manufacturer’s instructions. The RNA integrity and quantity of 3 samples were determined on an Agilent 2100 Bioanalyzer (Agilent, Shanghai, China). The library preparation, cluster formation, primer hybridization and sequencing reactions were performed sequentially according to the manufacturer’s recommended protocol. The quality of libraries was checked by Bioanalyzer 2100 (Agilent). The multiplexed libraries were sequenced using Illumina HiSeq2500 instrument at GENEWIZ Inc. in Suzhou, China.

### Read mapping and gene expression

In order to remove technical sequences, including adapters, polymerase chain reaction (PCR) primers, or fragments thereof, and quality of bases lower than 20, pass filter data of fastq format were processed by Cutadapt (version 1.9.1) to be high quality clean data and less than 75 bp sequences were then also removed after the trim. Due to no fully genome sequence information of *E*. *sinensis*, we used its draft genome sequences as a reference to map our sequencing clean reads. And gene model annotation files of relative species were downloaded from genome website (http://gigadb.org/dataset/100186). Hisat (v2.0.14) was used to index reference draft genome sequence [15]. Software TopHat (v2.0.9) was used to aligne clean data to reference genome and identify splice variants of each sample [16, 17]. Gene and isoform expression levels from the pair-end clean data were estimated using RSEM (v1.2.6). The sequences aligned with individual transcript were counted digitally. The expression levels for each gene were normalized to reads per kilobase of exon model per million mapped reads (RPKM) by the Htseq software (V 0.6.1) to facilitate the further analysis of transcripts [18, 19].

### Assembly and functional analysis of unigenes

Owing to no complete genome map, transcriptome de novo assembly was also performed to achieve more information about the gene expression in the *E*. *sinensis* developing testes [20]. The following analysis included Nr annotations, Swissprot annotations, Gene Ontology (GO) annotations, and analysis of Kyoto Encyclopedia of Genes, Genomes (KEGG) and Clusters of Orthologous Groups (COG) to identify the functions of unigenes and understand the distribution of gene functions in the immature testes, especially the genes related to the structures and the developmental stages of male germ cells.

### Validation of the unigenes

Some unigenes underwent real-time quantitative polymerase chain reaction (RT-qPCR) according to the manufacturer’s instructions to validate their expression in the developing testes and existence in the genome. The conditions of RT-qPCR reactions were: reaction mixture, 20 μl/tube; 95℃ 15 min, 1 cycle; 95℃ 10s, 60℃ 20s, 72℃ 10s, 40 cycles; 72℃ 5 min, 1 cycle. The internal control gene was 18S rRNA gene in this study. The primers for RT-qPCR were designed online using Primer-Blast (https://www.ncbi.nlm.nih.gov/tools/primer-blast/) and synthesized by Genecore Inc. (Suzhou, China). The primer sequences of RT-qPCR reactions were showed in S1 Table.

### The comparison of the unigenes in the developing testes with relevant data in databases

There are some data pertaining to the transcriptome and EST in the mature *E*. *sinensis* testes in NCBI database and other databases. Therefore, we further compared our unigenes with them.

## Results

### Transcriptome sequencing output, assembly and expression annotation

The Illumina HiSeq2500 sequencing of three replicons of samples produced a total of 145.19 M clean reads representing with a total of 21.34 Gb bases and 45.48% GC content (Table 1) and were assessed for quality. The results showed that our data had abundant sequencing depth. Average clean read size and Q20 percentage were 146.98 bp and 96.72%, respectively. There were 56.30% clean reads mapped the draft genome of *E*. *sinensis* (Table 2). 43.70% of the clean reads could not be matched to draft genome since there is not complete genome information for this species. Those results were consistent with the estimated percentage (55%-60%) of the draft genome to completed one.

**Table 1:** The data of raw and clean reads of RNA-seq.

Data	Samples	Length of reads	Number of reads	Total bases	Q20 (%)	Q30 (%)	GC (%)	N (ppm)
Raw data	XC-1	150	44574008	6686101200	95.42	90.86	45.98	152.06
	XC-2	150	41862256	6279338400	95.49	90.9	44.81	158.71
	XC-3	151	61327872	9260508672	95.96	91.22	45.84	9.38
Clean data	XC-1	147.26	43687506	6433501906	96.58	92.33	45.89	26.78
	XC-2	147.35	41128640	6060228931	96.58	92.27	44.71	26.13
	XC-3	146.57	60375298	8849167095	96.92	92.46	45.72	3.40

**Table 2:** Clean reads mapped the draft genome of *E*. *sinensis*.

Samples	Total reads	Total mapped	Multiple mapped	Uniquely mapped	Read1	Read2	Non_splice reads	splice reads	Reads mapped in proper pairs
XC-1	43687506	24059499 (55.07%)	2548157 (5.83%)	21511342 (49.24%)	11003608	10507734	15704969	5806373	17548280
XC-2	41128640	23020164 (55.97%)	2610035 (6.35%)	20410129 (49.63%)	10444057	9966072	15135012	5275117	16952110
XC-3	60375298	34658433 (57.41%)	4088691 (6.77%)	30569742 (50.63%)	15485261	15084481	21486995	9082747	27543734

As for assembly of the transcriptome, all clean reads were used to assemble contigs and resulted in 5691802 sequences, with a median length of 92 bp and 43.62% GC content. There were 406527 sequences obtained among all unigenes, with a median length of 406 bp and 43.19% GC content (Table 3). Total 24246 and 40793 unigenes were annotated using Swissprot and Nr database, respectively. Four unigenes were randomly picked out and amplificated using RT-qPCR. The result showed that they were genes existing in the genome of *E*. *sinensis* and also expressed in the developing testes (S1 Fig). Those resulted resource could be used to further study the functions of genes and genes’ products.

**Table 3:** The numbers of contigs and unigenes of the transcriptome assembly.

Type	sequences	Bases	Min	Max	Average	N50	(A+T)%	(C+G)%
All_Contig	5691802	433639972	25	19657	76.19	92	56.38	43.62
All_Unigene	406527	172440025	201	14733	424.18	406	56.81	43.19

**Fig 1:**
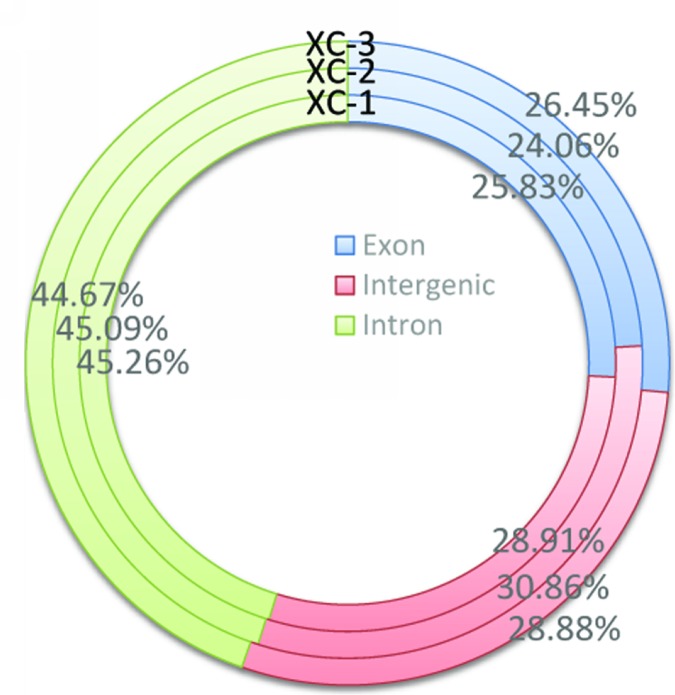
The distribution of reads mapped the exon, intron, and intergenic areas of draft genome of *E*. *sinensis*.

The statistic result total mapped reads matching to draft genome showed that the matching percentages of the reads to the exon, intron, and intergenic regions were 25.59%, 44.96% and 29.45%, respectively (Fig 1). Due to no completed genome information, the percentages of intron and intergenic regions were very high. It also indicated that there were a lot of genes expressing in the developing testes which functions were not revealed using this statistic method. Therefore, we assembled and annotated the transcriptome as mentioned above. And then, the resulting unigenes underwent the analysis of GO, COG, and KEGG.

**Fig 2:**
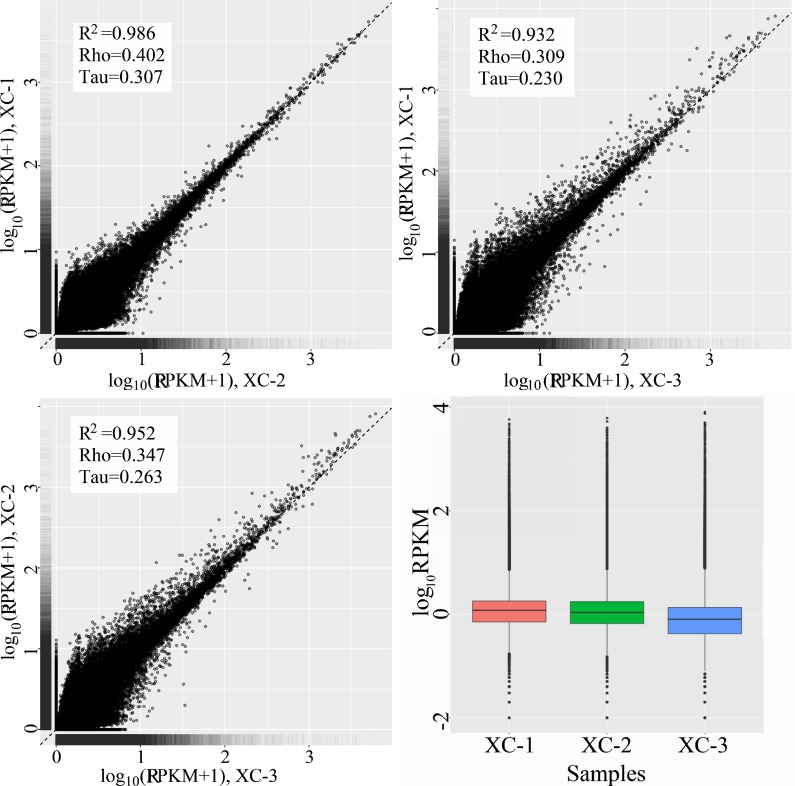
The pearson correlation and RPKM Box plots of gene expression in the replocons of 3 RNA-seq samples. The maximum, upper quartile, median, lower quartile, and minimum from top to down in the RPKM Box plots.

We also analyzed both the Pearson correlation and the RPKM distribution of gene expressing levels between replicons of RNA-seq samples. Those results showed that our data were of high quality and well reproducible (Fig 2).

### Genes involved in spermatogenesis and testis development

**Fig 3:**
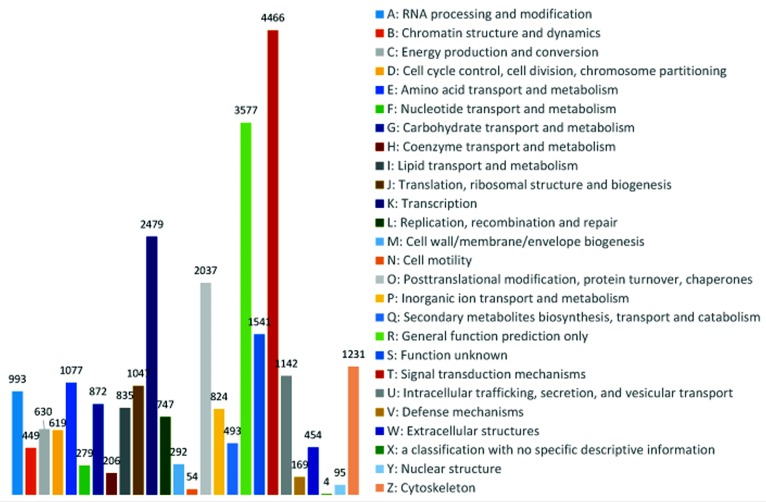
Histogram presentation of clusters of orthologous groups (COG) classification. A: RNA processing and modification; B: Chromatin structure and dynamics; C: Energy production and conversion; D: Cell cycle control, cell division, chromosome partitioning; E: Amino acid transport and metabolism; F: Nucleotide transport and metabolism; G: Carbohydrate transport and metabolism; H: Coenzyme transport and metabolism; I: Lipid transport and metabolism; J: Translation, ribosomal structure and biogenesis; K: Transcription; L: Replication, recombination and repair; M: Cell wall/membrane/envelope biogenesis; N: Cell motility; O: Posttranslational modification, protein turnover, chaperones; P: Inorganic ion transport and metabolism; Q: Secondary metabolites biosynthesis, transport and catabolism; R: General function prediction only; S: Function unknown; T: Signal transduction mechanisms; U: Intracellular trafficking, secretion, and vesicular transport; V: Defense mechanisms; W: Extracellular structures; X: A classification of KOG annotations on the NCBI, with no specific descriptive information and only one X class; Y: Nuclear structure; Z: Cytoskeleton.

**Fig 4:**
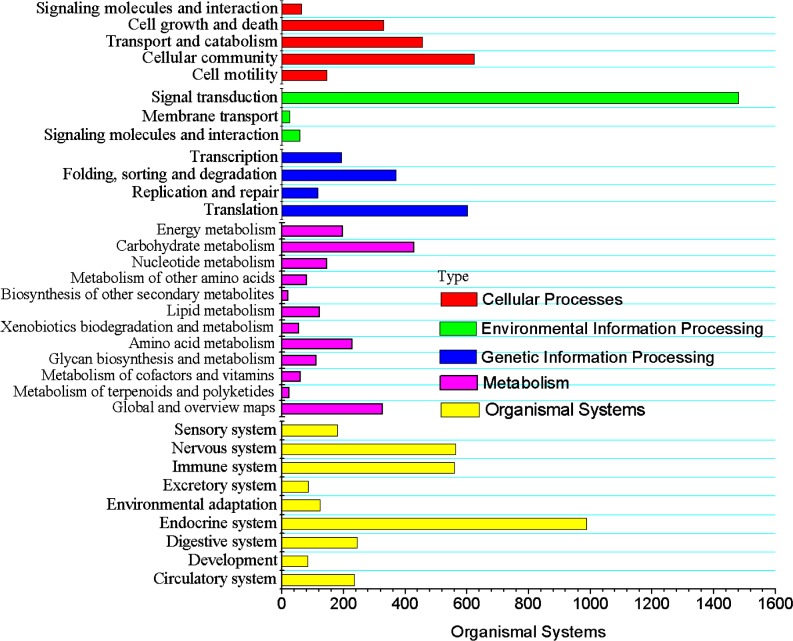
All unigene KEGG barplot about pathways, categories, and count.

The analytic results of KOG, GO and KEGG showed wide potential molecular functions of transcripts in the developing testes (Figs 3 and 4). KEGG analysis of unigenes yielded total 9422 predicted genes. Those predicted genes were involved in total 216 KEGG pathways related to the physiological activities of developing testes such as cellular processes, genetic information processing, metabolism, organismal systems and environmental information processing, and organismal systems (Fig 4 and Table 3). The top ten function of gene number were signal transduction (1635), endocrine system (988), cellular community (623), translation (602), nervous system (564), immune system (560), transport and catabolism (456), carbohydrate metabolism (428), folding, sorting and degradation (369), and cell growth and death (330). Our particular interest was the structural traits pertaining to spermatogenesis. Those traits involved 23 pathways pertaining to cellular and subcellular structural alteration of male germ cells during the cell division and proliferation, developmental differentiation, maturation, apoptosis, and the degradation of cellular components in our analytic results. There were five events related to the cell division and proliferation. They were signaling pathways regulating pluripotency of stem cells (ko04550), cell cycle (ko04110), cell cycle–yeast (ko04111), meiosis–yeast (ko04113), and cellular apoptosis (ko04210). Another eleven events were associated to development, differentiation, and maturation. They were ribosome biogenesis in eukaryotes (ko03008), ribosome (ko03010), proteasome (ko03050), cytokine-cytokine receptor interaction (ko04060), focal adhesion (ko04510), ECM-receptor interaction (ko04512), cell adhesion molecules (CAMs) (ko04514), adherens junction (ko04520), tight junction (ko04530), gap junction (ko04540), and regulation of actin cytoskeleton (ko04810). The last seven events connected to degradation of cellular components. They were ubiquitin mediated proteolysis (ko04120), regulation of autophagy (ko04140), lysosome (ko04142), endocytosis (ko04144), phagosome (ko04145), and peroxisome (ko04146). Interestingly, we also found 583 unigenes which might be involved in the decondensation of spermatozoal nuclei. Most of them were not found to take part in the pathways of KEGG. Their function relevant to decondensation will be analyzed in the section of discussion. These predicted events provide a view of the potential molecular mechanisms of spermatogenesis in *E*. *sinensis*.

### Similarity but difference of immature testis unigenes compared with the mature

Compared to the data of *E*. *sinensis* testis in the Nr database, there were 48213 unigenes (70.31%) with identity of more than 99 matching to 68570 mature testis unigenes. Another comparison is our unigenes with the data in EST, which presented 7858 unigenes (46.25%) with identity of more than 99 matching to16987 ESTs of *E*. *sinensis*. As for the pathways, we found that many in the mature testes of *E*. *sinensis* also appeared in the developing testes. The examples were firstly in basic substance and energy metabolism, such as purine metabolism and pyrimidine metabolism. The others were in the genetic information transmission, such as DNA replication and the various ways of reparation of misreplication, transcription, spliceosome. The third types of examples were in the cell division, such as mitotic proliferation of spermatogonia, meiosis, recombination, cell cycle regulation. The fourth types of examples presented in the cell construction and remodeling, such as focal adhesion, adherens junction, and regulation of actin cytoskeleton, endocytosis, phagosome, ubiquitin-mediated proteolysis, and Fc gamma R- mediated phagocytosis. In addition, some important pathways, such as chemokine signaling pathway, MAPK signaling, hormone biosynthesis, steroid hormone biosynthesis, apoptosis, p53 signaling pathway, calcium signaling pathway, Wnt signaling pathway, and peroxiredoxin involved in anti-oxidant system, other anti-hyperthermia stress, and anti-oxidative stress pathways, also appeared in both the mature and the immature testes. In addition to same events in both types of testes, we also found some different ones appeared in the developing testes or presented different genes in diversity pathways mentioned above, such as phagosome, mitotic proliferation of spermatogonia, regulation of actin cytoskeleton, ubiquitin-mediated proteolysis, p53 signaling pathway, other anti-hyperthermia stress, anti-oxidative stress pathways, and so on. Those comparison indicated that the transcriptome of developing testes in *E*. *sinensis* was similar to one of the mature but yet presented some different traits.

### Data deposition

The data from the developing testis in *E*. *sinensis* were deposited in the National Center for Biotechnology Information (NCBI, USA, http://www.ncbi.nlm.nih.gov/) with accession number: SRR5054211, SRR5054212, and SRR5054223.

## Discussion

Spermatogenesis is an essential biological event and a key link of generation alternation between diploid and haploid in the reproduction of most animals. By spermatogenesis, diploid somatic cells are altered into haploid sexual cells. For most animals, meiosis is the most typical physiological process during the spermatogenesis. However, the structures of spermatozoa produced by different animals are diverse because of the differences of both phylogenetic environment and genetic background. Particularly, it is worth focusing on both the differences in the compact state of spermatozoal genetic material and various molecular mechanisms to establish these different states. Unlike mammalian, avian and fish, which have the spermatozoa with condensed or semi-condensed nuclei, the spermatozoa of *E*. *sinensis* have typical decondensed nuclei. However, the mechanism of nuclear decondensation of latter yet needs to further study although some roles of many genes and (or) their encoding proteins involved in the spermatogenesis of *E*. *sinensis* had been reported [4–9, 21–27]. The transcriptome traits of developing testes would give us a window to look at the tip of the iceberg of the relevant issue. By RNA-seq, we harvested transcriptome data of developing *E*. *sinensis* testes with both a high-quality and a sufficient sequencing depth. Further assembly and comparative analysis showed that among a total of 145.19 M clean reads representing with a total of 21.34 Gb bases 56.30% of them was matched to the draft genome of *E*. *sinensis*. The draft genome would be mapped about 55%-60% of the complete genome of this species. By assembly of the transcriptome, we achieved contigs of 5691802 sequences and unigenes of 406527 sequences. Among them, 9422 predicted genes were involved in total 216 KEGG metabolic pathways. Compared to the mature testis unigenes of *E*. *sinensis*, the developing testes presented similar transcriptome, however, also appeared some different traits. It was particularly interesting that some events were involved in the cellular and subcellular structural alteration of male germ cells during the cell division and proliferation, developmental differentiation, maturation, apoptosis, and the degradation of cellular components. We here focused on the genes involved in those events during the spermatogenesis in *E*. *sinensis*.

### The cell division and proliferation of male germ cells

We found five events specifically related to the cell division and proliferation, including signaling pathways regulating pluripotency of stem cells, cell cycle, cell cycle–yeast, meiosis–yeast, and apoptosis in the transcriptomic data. In the testis, spermatogonial stem cells (SSCs) are basic cells with an indefinite self-renewal capacity and the potential to generate both new SSCs and spermatogonia through mitosis [28–30]. The diploid spermatogonia then undergo meiosis to generate haploid spermatids. The spermatids further differentiate into mature spermatozoa. This serial process from spermatogonia to spermatozoa is called spermatogenesis. The SSCs in the testis belong to induced pluripotent stem (iPS) cells, one of two types of pluripotent stem cells (PSCs). The iPS cells have defined reprogramming factors, Oct4, Sox2, Klf4 and c-Myc. Those factors determine the pluripotency of iPS cells by the signaling pathways regulating pluripotency of stem cells with other relevant signal molecules [28–30]. The result in the present study showed that there were 48 signal molecules involved in the signaling pathways regulating pluripotency of stem cells. Those signal molecules were Activin, Wnt, and their downstream targeting proteins, such as ACVRI/II, Frizzled, and so on. These signaling pathways were able to coordinately promote self-renewal and pluripotency [30–32]. This pluripotency endued the testis to a capacity that the testis can continuously generate new SSCs and a number of spermatozoa.

The way of generation of new SSCs and spermatogonia is mitosis. Mitotic cell cycle includes G1, S, G2 and M phases. Both RNAs and proteins are synthesized at the G1 and G2 phases and DNA replication occurs at the S phase. M phase refers to mitosis process which can be divided into 4 phases: prophase, metaphase, anaphase, and telophase. Cyclin-dependent kinases (CDKs), E2F, rRb, cyclin-CDK inhibitors (CKIs) are involved in the pathway through which the cell cycle is regulated [33–36]. p53 and its transcriptional targets determine both G1 and G2 checkpoints [37, 38]. Intra-S-phase checkpoint can be activated through ATR-Chk1-mediated protein degradation of Cdc25A protein phosphatase [39, 40]. In yeast Cdc28 association with Clb2 and Clb1 promotes entry into mitosis [41, 42]. In our study, we also found 139 predicted genes were associated to the mitosis. It indicated that the regulatory pathways of cells in the other organisms also play essential roles in the regulation of development of immature testes and the spermatogenesis in *E*. *sinensis*.

The spermatogonia generated through mitosis would undergo meiosis which is a main event during the spermatogenesis in the testis. Meiosis of a spermatogonium results in two primary spermatocytes at meiosis I which further divide into four secondary spermatocytes at meiosis II. The latter then alter into spermatids. Nutritional signals can control whether yeasts enter meiosis phase [43, 44]. We found that 57 predicted genes in those signaling pathways presented in the developing testes, such as Gpr1, PKA, Glc7, Mad2, APC/C, Cdc20, ESP1, Smc1, Smc3, In1, MCM, Rts1, PP2A, and so on [45–48]. Those signal molecules take part in determination of the DNA replication, arm cohesion of sister chromatids, or meiotic chromosome segregation. The results demonstrated that nutritional signals played an important role during the spermatogenesis in the testes.

### The development, differentiation, and maturation of male germ cells

In *E*. *sinensis*, there are five types of dominant male germ cells distributing in the developing testis: spermatogonia, primary spermatocytes, secondary spermatocytes, spermatids, and spermatozoa. After the meiosis II, the secondary spermatocytes further alter into spermatids. The morphology and structure of spermatids then change and finally become to mature spermatozoa. Those different types of cells in turn located in the epithelium of from the distal to proximal seminiferous tubule or disperse in the lumen of seminiferous tubule. Especially, both the spermatids and the immature spermatozoa are embedded in the meshes formed by nutrient cells, one spermatid or immature spermatozoon in each mesh. The nutrient cells provide nutrient to the spermatids or the immature spermatozoa surrounded by them through gap junctions or other tunnels. The spermiogenesis also involve the remodeling the cellular inner and outer structure, especially the process of maturation of spermatozoa. Each mature spermatozoon in *E*. *sinensis* has a cup-shaped nucleus and about 20 radial arms derived from the nuclear membrane. Radial arms contribute to the maintenance of the state of spermatozoon, especially the cup-shaped state of nucleus and maybe facilitate to directly substance exchange and signal transduction of nucleus with its surroundings [1]. Those roles of radial arms can facilitate to the recognition and fusion of spermatozoon with egg.

There are many ways, such as cellular junction, matrix proteins, and cytoskeleton proteins, which take part in the processes of development, differentiation, and maturation of cells and tissues. For example, actin is one of most important cytoskeleton proteins in *E*. *sinensis* spermatozoa and also a signaling molecule. It plays important roles in the male reproduction in *E*. *sinensis* [11]. Our results showed that 744 predicted genes related to those processes presented in the developing testes in *E*. *sinensis*. Those predicted genes were involved in some pathways such as cytokine-cytokine receptor interaction, focal adhesion, ECM-receptor interaction, cell adhesion molecules (CAMs), adherens junction, tight junction, gap junction, and regulation of actin cytoskeleton. In addition, another 397 predicted genes involved in the ribosome biogenesis in eukaryotes or composition of ribosome presented in the developing testes. This information can better the understanding of the mechanism of spermatogenesis in *E*. *sinensis*. For example, the signal molecules involved in gap junction, such as RTK, TUBA, TUBB, Ras, MEK1/2, CDC2, Gi, Gq, ADCY, PLC, PKA, PKC, IP3R, and so on, presented in the developing testes indicated that the gap junctions were an intercellular channels during the spermatogenesis. Many male germ cells and their adjacent cells can direct communicated between their cytosolic compartments. It enhances the efficiency and capacity of male germ cells to obtain small molecules including ions, amino acids, nucleotides, second messengers and other metabolites from the adjacent cells, thus contributing to the spermatogenesis. Those physiological events also play critical roles in the metabolic transport, apoptosis, and tissue homeostasis. The event of ribosome biogenesis in eukaryotes was another event during the testis development and spermatogenesis in *E*. *sinensis*. The spermatogenesis needs plenty of proteins which can be made in ribosomes. Therefore, ribosome biogenesis in the developing testes in *E*. *sinensis* was consistent with this need.

The late stage of spermatogenesis, the process from spermatid to mature spermatozoon, is called spermiogenesis. During the spermiogenesis, the spermatid format an acrosome surrounded by a cup-shaped nucleus. In our study, we found many actin and actin-binding proteins exist in *E*. *sinensis* immature testes. This finding is similar to that of Wang et al. in *E*. *sinensis* mature testes [11]. The function of those actin cytoskeleton, same as in the mature testes, should play a key role in the acrosome formation [11].

### The decondensation of spermatozoal nuclei

There is a typical decondensed nucleus in the *E*. *sinensis* spermatozoon. The decondensation of chromatin appears in the mid-spermatid. Many factors determine the compactness state of the chromatin, such as histones and histone binding proteins, modification of histones, DNA modification and DNA binding proteins. Recent research has found that the acetylation and the decrease of histones maybe contribute to the decondensation of chromatin [9]. Our results showed that 583 predicted genes might relate to the decondensation of chromatin in spermatozoal nucleus in *E*. *sinensis* although most of them were not found involved in the pathways of KEGG. We categorized the products of those predicted genes into 4 types: (1) proteins related to modification of chromatin, (2) histones, histone binding proteins, and proteases related to modification of histones, (3) DNA binding proteins and proteins related to DNA modification, (4) nuclear distribution protein NudE. We found three kinds of predicted genes of proteins related to modification of chromatin. First were chromodomain-helicase-DNA-binding proteins (CHD4 and MI2B, CHD5, CHD6 and CHD9). Second were DNA topoisomerases (TOP1, TOP2, and TOP3). Third were apoptotic chromatin condensation inducers in the nucleus (ACIN1, ACINUS). Those proteins related to modification of chromatin can loosen the structure of chromatin [49]. We also found core histones (H2A, H2B, H3, and H4), histone binding proteins (RBBP4, HAT2, CAF1, MIS16), and proteases related to modification of histones. The later included histone deacetylase (HDAC1_2, HDAC10, HDAC11, HDAC3, HDAC4_5, and HDAC6) and lysine-specific demethylase (KDM3, KDM6B, JMJD3, KDM6B, JMJD3, HR, and KDM1A, AOF2, LSD1). Both the histone binding proteins and the proteases related to modification of histones in our study could decrease the chance of interaction of histones with DNA, resulting in the DNA loose. There were 3 kinds of DNA binding proteins presented in the testes of the crab. First were high mobility group proteins (HMGN5, HMGB1, and HMGB2). Second were cellular nucleic acid-binding proteins (KIN, EMC, CNBP, CSRNP, and FPR3_4). Third were zinc finger proteins (BAZ2A, TIP5, ZKSCAN, KRAB, MZF1, RC3H, SCAN, ZBTB17, MIZ1, ZBTB43, ZBTB7, ZEB1_2, ZFHX2, GLI2, GLI3, DPF2, REQ) and class B basic helix-loop-helix protein 8 (BHLHB8, MIST1). Both the high mobility group proteins and the cellular nucleic acid-binding proteins are non-specific DNA binding proteins but can construct the nuclear skeleton. Complex nuclear skeleton does not compact the decondensed chromatin but can, to a certain extent, stabilize, maintain and protect chromatin. The zinc finger proteins and class B basic helix-loop-helix protein 8 are specific DNA-binding proteins. They can stabilize, maintain and protect DNA helical structure [50]. As for proteins related to DNA modification, DNA methyltransferase 1-associated proteins (DMAP1, SWC4, and EAF2) were found. They are involved in the DNA methylation [51]. The nuclear distribution protein NudE (NDE1, NUDE) also might play an important role in decondensed nuclear migration [52]. In addition, there were a large number of nuclear factors (NFIA, NFRKB, INO80G) and nuclear receptor subfamily (NR1D3, NR1F4, NR4A2, NURR1, NR5A2, FTF, K08709). They also are specific DNA-binding proteins, which contain the information of nuclear location [53, 54]. Those genetic and epigenetic factors might contribute to the decondensation and stability of decondensed nuclei in the *E*. *sinensis* spermatozoa.

### The degradation of components of male germ cells

The degradation of cellular components can remove the misfolding, misfounctional, redundant or “aging” biomacromolecules or degenerated, damaged organelles in living cells through regular program to maintain the normal physiological activity of cell. During the spermatogenesis where the metabolism is considerably active, the degradation of cellular components is common. In the present study, 563 predicted genes pertaining to degradation of cellular components were found in the developing testes, such as F-actin, vATPase, Rab7, Dynein, TUBA, TUBB, cathepsin, Sec61, Rac, and so on. They were participants of proteasome, ubiquitin mediated proteolysis, regulation of autophagy, lysosome, endocytosis, phagosome, or peroxisome. There are specific receptors on the surface of some types of cells, which can recognize ligands on the surface of relatively large particle and take the particle to form phagosome [55–57]. This process is named phagocytosis. Phagocytosis is a key mechanism in the tissue remodeling besides in the defense against infectious agents [58–60]. The phagosome needs to fuse lysosomes or other membrane organelles, such as endosomes to degrade the particles engulfed by it into fragments [59, 60]. More than 40 hydrolases in acidic lysosome are packaged into clathrin-coated vesicles and are transported to late endosomes [61–64]. Substances in them then were digested via endocytosis, phagocytosis, and autophagy. In the present study, 563 predicted genes, such as GNPT, AP-1, AP-3, LGMN, NAGA, GALC, GUSB, HEXA/B, MANB, LAMAN, SGSH, NPC, NRAMP, ABCA2, and so on, were involved in degradation of cellular components through the phagosome, lysosomes, and other relevant membrane organelles. During the spermiogenesis in *E*. *sinensis*, the spermatid dramatically change its shape and structure, especially emerging small vesicles in their cytoplasm to finally merge into a large proacrosomal vesicle. The latter terminally becomes a round-shaped mature acrosome. It is believed that the acrosome originates from the Golgi apparatus and is a specialized lysosome. To date, the mechanism of acrosomal formation in the *E*. *sinensis* spermatozoa is yet unclear. Our findings suggested that those membrane organelles were likely to take part in the process of acrosomal biogenesis and reconstruction of cell shape and structure.

In conclusion, the transcriptomic analyses of developing testes in *E*. *sinensis* identified many candidate genes potentially involved in development of testes and spermatogenesis in this species. Those predicted genes were involved in total 216 KEGG metabolic pathways, especially ones associated to cellular and subcellular structural alteration of male germ cells during the cell division and proliferation, developmental differentiation, maturation, apoptosis, and the degradation of cellular components during the spermatogenesis in *E*. *sinensis*. The unigenes encoding the genetic and epigenetic factors also were found, which might contribute to the decondensation and stability of decondensed nuclei in the spermatozoa. The functions of those predicted genes are coming to further study to understand the molecular basis of development of testes and spermatogenesis in this species.

## Supporting information

S1 Table The primer sequences of RT-qPCR. (PDF)

S1 Fig The validation of unigenes using RT-qPCR. (PDF)
