# Supplementary material for: Transcriptome using Illumina sequencing reveals the traits of spermatogenesis and developing testes in Eriocheir sinensis
Source: PLoS One. 2017 Feb 17;12(2):e0172478. doi: 10.1371/journal.pone.0172478 (PMC5315355; doi:10.1371/journal.pone.0172478)
Supplement: S1 Table — (PDF) [file pone.0172478.s001.pdf]

S1 Table. The primer sequences of RT-qPCR.

Gene ID	Names of primers	Sequences of primers
GU362670 (18S ribosomal RNA gene)	GU362670F	TGATTACGTCCCTGCCCTT
GU362670 (18S ribosomal RNA gene)	GU362670R	ACATCTTTCCGTCAGCTCG
JR777485	JR777485F	CCTTCTTGGCGGCCTTTCCT
JR777485	JR777485R	AACATTCGCTCGCCGTCTGG
KA667799	KA667799F	GCACTACAGCCCATACCGAG
KA667799	KA667799R	GGTCTTCATCCCGTCTCCTG
JR765792	JR765792F	CTCAGGCAGCGGCATTAACC
JR765792	JR765792R	CCTACACTGAGCACGCCAAG
KA663272	KA663272F	TGCCCAGAATCGTCCAGAGT
KA663272	KA663272R	GCAACACTGCCGTCTCATCT
