# Supplementary material for: Transcriptome using Illumina sequencing reveals the traits of spermatogenesis and developing testes in Eriocheir sinensis
Source: PLoS One. 2017 Feb 17;12(2):e0172478. doi: 10.1371/journal.pone.0172478 (PMC5315355; doi:10.1371/journal.pone.0172478)
Supplement: S1 Fig — (PDF) [file pone.0172478.s002.pdf]

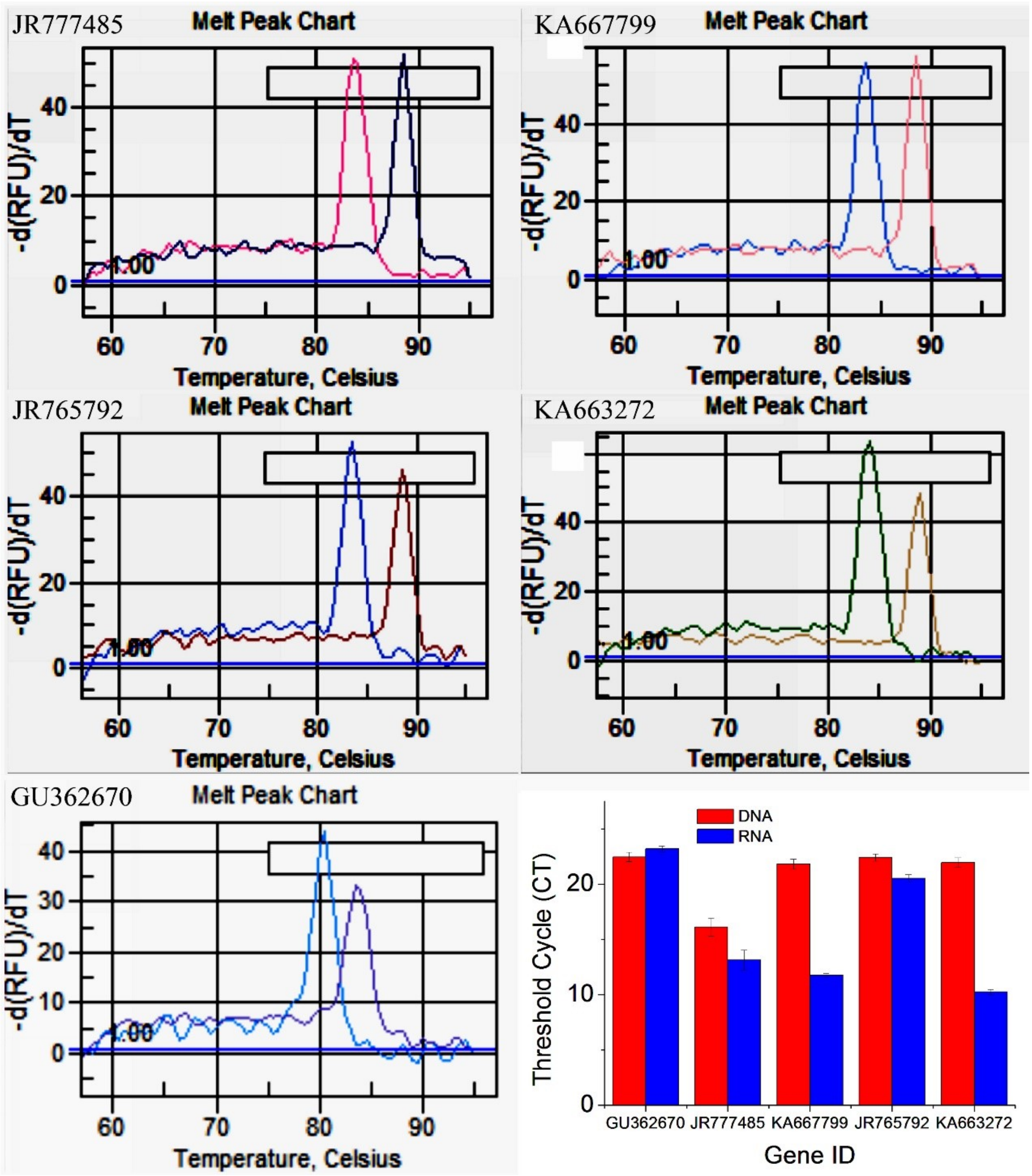

S1 Fig. The validation of unigenes using RT-qPCR. Four unigenes (JR777485, KA667799, JR765792, and KA663272) and one 18S rRNA gene (GU362670) using RT-qPCR (n = 3). The result showed that they existed in the genome (second peak in each melt peak chart) and also expressed in the developing testes (first peak in each melt peak chart) of *E. sinensis*.
